# Complex Evolutionary Dynamics of H5N8 Influenza A Viruses Revealed by Comprehensive Reassortment Analysis

**DOI:** 10.3390/v16091405

**Published:** 2024-09-03

**Authors:** Egor Degtyarev, Sofia Feoktistova, Pavel Volchkov, Andrey Deviatkin

**Affiliations:** 1 Federal Research Center for Innovator and Emerging Biomedical and Pharmaceutical Technologies, 125315 Moscow, Russia; 2 Center for Personalized Medicine, The MCSC Named after A.S. Loginov, 111123 Moscow, Russia

**Keywords:** H5N8, reassortment, phylogeny

## Abstract

Influenza A viruses (IAVs) circulate among different species and have the potential to cause significant pandemics in humans. This study focuses on reassortment events in the H5N8 subtype of IAV, which poses a serious threat to public health due to its high pathogenicity in birds and potential for cross-species transmission. We retrieved 2359 H5N8 IAV sequences from GISAID, and filtered and analyzed 442 complete genomic sequences for reassortment events using pairwise distance deviation matrices (PDDMs) and pairwise distance correspondence plots (PDCPs). This detailed case study of specific H5N8 viruses revealed previously undescribed reassortment events, highlighting the complex evolutionary history and potential pandemic threat of H5N8 IAVs.

## 1. Introduction

Influenza viruses are a group of viruses that circulate in various species, including birds [1], pigs [2], bats [3], dogs [4], cats [4,5], marine mammals [6], and humans. These viruses cause seasonal epidemics in humans, leading to 300–500 thousand deaths each year [7]. Influenza viruses are divided into four species: A, B, C, and D. It is noteworthy that influenza A viruses are responsible for most of the major human pandemics [8].

Influenza A viruses (IAVs) form particles, with the spherical forms having a diameter of about 100 nm and the filamentous forms often exceeding 300 nm in length [9]. According to the International Committee on Taxonomy of Viruses (ICTV) IAVs belong to the order *Articulavirales* and the family *Orthomyxoviridae* (accessed on 1 December 2023) [10]. The natural hosts of the IAVs are wild birds [11].

The influenza genome consists of eight segments of single-stranded viral RNA with a negative sense [12]. There are three segments coding for the viral RNA-dependent RNA polymerase—PB1, PB2, PA. Other segments encode nucleoprotein (NP), matrix protein (M1) and membrane protein (M2), nonstructural protein (NS1), and nuclear export protein (NEP). The remaining segments, which code for glycoproteins that envelop the virus and influence its antigenic behaviour, are called hemagglutinin (HA) and neuraminidase (NA).

The HA and NA genes of IAVs are the most variable and play an important role in the life cycle of the virus, which is why the influenza A virus has been divided into subtypes based on these genes. There are 19 different HA genes and 9 different NA genes, and each IAV is classified according to these genes [13,14].

There are several characteristics that contribute to the rapid evolution and great diversity of viruses, such as having a large viral population size, short generation times, and high mutation rates. Reassortment is thought to be one of the main reasons for the emergence of new viruses with pandemic potential. The H2N2 virus, for example, which emerged in 1957 (H2N2/1957) and caused the “Asian flu pandemic”, arose through reassortment between the previously circulating human H1N1 virus and avian viruses [15,16]. Reassortment, which is facilitated by the segmented structure of the influenza genome, occurs when two different strains coinfect a single cell and exchange intact genome segments [17]. In the case of the coinfection of two IAVs with eight segments each, a total of 256 unique progeny genotypes can potentially be generated. This process significantly increases the diversity of the virus and promotes rapid evolution under selective pressure [18]. The reassortment process includes adaptation to new host environments, the evasion of host immune responses, and the acquisition of resistance to antiviral drugs. An example of such an adaptation is H1N1pdm2009, which shows some evidence of a reassortant form [19].

Highly pathogenic avian influenza viruses (HPAIs) pose a serious threat to public health [20]. These viruses, which are highly contagious among birds, can also infect humans. There is currently no vaccine for these subtypes, although research is underway to develop one. It is possible that humans may come into contact with birds carrying HPAI viruses that could potentially lead to a pandemic. H5N8 IAVs are considered a possible trigger of a new pandemic [21,22,23].

An HPAI H5N8 virus with a segment encoding the hemagglutinin (HA) surface protein of the H5N1 subtype, A/Goose/Guangdong/1/1996, was first detected in birds at live bird markets in China in 2010 [24]. This HPAI H5N8 virus has acquired the HA gene segment of the HPAI H5N1 virus and other gene segments from several other AIVs circulating in eastern China through reassortment, and is now categorized as being in HPAI H5 virus clade 2.3.4.4 [24]. This clade is unusually promiscuous and has been found in combination with six different neuraminidase (NA) segments, allowing multiple H5Nx viruses to circulate at the same time and in the same region [22,23]. The propensity of HPAI H5 virus clade 2.3.4.4 to form novel subtypes that can spread rapidly and globally is of great concern. The HPAI H5N8 virus caused a large outbreak of avian influenza in poultry in South Korea in the winter of 2013–2014, and subsequently spread to Japan, North America, Europe, and Russia, causing outbreaks there between fall 2014 and spring 2015 [24,25,26,27]. There is some evidence of the transmission of HPAI H5N8 from birds to mammals. During the fall/winter season of 2020/21, there were extensive outbreaks of highly pathogenic avian influenza (HPAI) in Europe [28]. The H5N8 subtype has predominated both in wild birds and domestic poultry, in which there have been outbreaks of this subtype. In the UK, there have been 22 outbreaks of H5N8 in domestic poultry and captive birds, and more than 300 detections of H5N8 in wild birds from the autumn/winter period of 2020/21 to April 2021. In addition, H5N8 of avian origin has been detected in five swans, one fox, and three seals at a wildlife rehabilitation centre [29]. At the same time, the same IAV strain was detected in harbour seals on the German North Sea coast [30].

Rassortment can regularly give rise to variants of the H5N8 influenza virus that pose a pandemic threat and could lead to the emergence of new variants. Non-HA and NA segments may be taken over by other viruses that are less pathogenic and, therefore, not effectively monitored. Although many reassortant H5N8 viruses are known, to our knowledge there is no information on a systematic analysis of reassortment events in all representatives of this subtype. Given this background, we conducted a comprehensive analysis of reassortment events in the H5N8 influenza virus subtype to uncover its patterns and gain new findings.

## 2. Materials and Methods

### Sequence Selection for Analysis

All available sequences of H5N8 IAVs were retrieved from GISAID in December 2023 (n = 2359).

All segment sequences were concatenated to the complete protein-coding regions for each virus using Virus Segment Concatenator [31]. Only complete protein-coding fragments of the sequences were used for further analysis. Sequences with degenerate nucleotides were omitted. Sequences that were nearly identical (more than 99.5% identical nucleotides) were excluded. The sequence A/UNL/HK/2:6/2017 (H5N8) was omitted as it is of artificial origin [32]. Finally, the dataset contained concatenated genomic regions of 442 viruses.

All available sequences of H5N8 IAVs were retrieved from GISAID in December 2023. A total of 26,405 sequences were downloaded (Supplementary Table S1, sheet “Sequences retrieved from GISAID”). In this case, only those viruses were selected for which sequences of all segments were available according to the database. It should be noted that individual segments of some viruses were duplicated in GISAID and had different sequence identifiers. In this case, only the viruses for which the sequences of all segments were available in the database were selected. It should be noted that individual segments of some viruses were duplicated in the database and had different sequence identifiers. For example, there were 10 sequences for eight segments of virus A/quail/Korea/H551/2020 (Supplementary Table S1, sheet “Sequences retrieved from GISAID”). For such viruses, only one of the duplicated segments was used for the further analysis steps.

All segment sequences were concatenated to the complete protein-coding regions for each virus using Virus Segment Concatenator [31]. Only the complete protein-coding fragments of the sequences were used for further analysis. A total of 2359 H5N8 viruses with concatenated genomes were generated using Virus Segment Concatenator. It should be noted that this tool works correctly but depends on the uniform name given in the Fasta header. In this study, all Fasta headers were standardized in the following format—“Identifier(H5N8)_segment_number|Isolate ID” (for example, “A/duck/Czech_Republic/7681-5/2021(H5N8))_segment_5|EPI_ISL_1941480”).

Sequences with degenerate nucleotides were omitted using in-house scripts. Sequences that were nearly identical (more than 99.5% identical nucleotides) were excluded from the analysis using CD-HIT software version 4.8.1 [33]. The sequence A/UNL/HK/2:6/2017 (H5N8) was manually omitted from the dataset as it is of artificial origin [32]. Finally, the dataset contained concatenated genomic regions of 442 viruses (Supplementary Table S1, sheet “Analyzed viruses”). The multiple sequence alignment was performed with MAFFT version 7 [34], using the default settings (automatic selection of the most suitable alignment strategy, 200PAM/k = 2 scoring matrix, gap opening penalty 1.53, offset value 0.0).

Reassortment screening was performed using pairwise distance-deviation matrices (PDDMs) and pairwise distance-correspondence plots (PDCPs), as previously described in [35].

Simplot 3.5.1 was used to generate the similarity plots with the parameters: GapStrip: on, Kimura (2-parametr), T/t: 2,0 [36]. The visualization of the similarity plots was performed using CorelDraw.

Phylogenetic trees were created using the neighbour-joining method and the bootstrap test of phylogeny (n = 500), implemented in MEGA 11 [37]

## 3. Results

**Figure 1:**
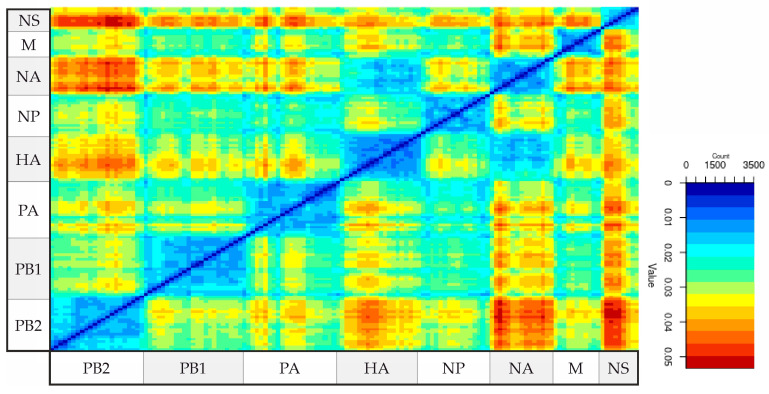
Pairwise distance-divergence matrix (PDDM) for H5N8 influenza viruses (window = 300 bp, step = 100 bp). The PB2 subunit, HA, NA, and NS segments were visually the most involved in reassortment events compared to the rest of the genome. The color gradient scale shows the root mean square error (RMSE) values in PDCP, constructed for the corresponding genomic regions using sliding window.

A total of 2359 sequences assigned to the H5N8 subtype were retrieved from GISAID. The complete sequences of the segments were sequentially concatenated into a single sequence for each of the 422 H5N8 viruses. The PDDM was generated for all possible pairs of genomic regions with a sliding window of 300 nucleotides and a step of 100 nucleotides (Figure 1). The root mean square error (RMSE) of all pairwise distances of two genomic regions from the regression line reflects the extent of phylogenetic incongruence between these fragments of the genome. This plot indicates, by the red colour, whether there was a high phylogenetic incongruence between the corresponding genomic regions, while a low phylogenetic incongruence between the corresponding genomic regions is indicated by the blue colour. A high RMSE indicates a possible reassortment between the most divergent viruses in the corresponding regions of the genome. The PDDM showed that reassortment between the most divergent viruses occurred between the PB2, HA, NA, and NS segments (shown in red in Figure 1).

**Figure 2:**
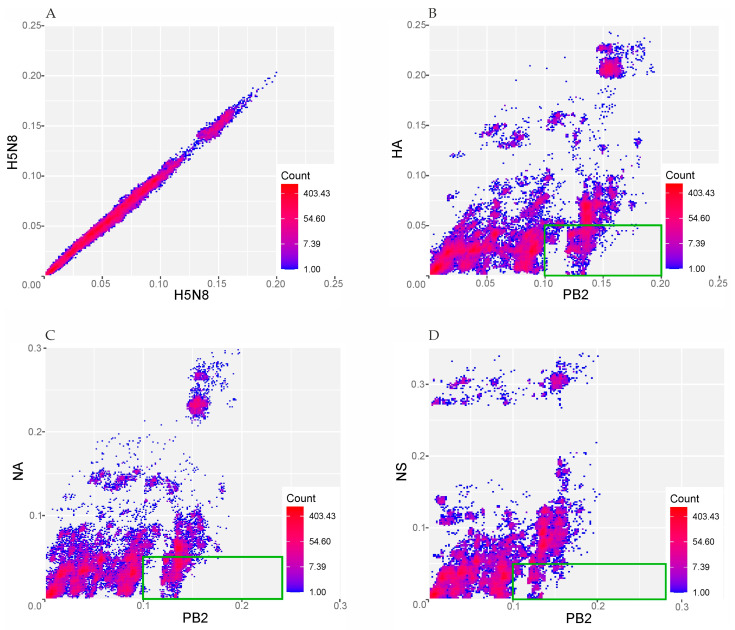
Pairwise nucleotide distance comparison plots (PDCPs) showing phylogenetic incongruence between selected genetic regions of H5N8 influenza viruses. Each dot represents a pair of raw nucleotide distances between two sequences in two genomic regions (axis labeling). The count value indicates the number of virus pairs per bin by a color gradient. The region of subsequent analysis was highlighted with the green boxes. (**A**) PDCP constructed for the concatenated full genomic sequences of H5N8 viruses for which pairwise distances were calculated for even and odd sites (negative control); (**B**) PDCP constructed for PB2 and HA segments of H5N8 influenza viruses; (**C**) PDCP constructed for PB2 and NA segments of H5N8 influenza viruses; (**D**) PDCP constructed for PB2 and NS segments of H5N8 influenza viruses.

PDCPs were generated for the PB2, HA, NA and NS segments to show differences in the pairwise evolutionary distances between these genomic regions (Figure 2). Briefly, the percentages of different nucleotides between the PB2 and HA segments (Figure 2B), PB2 and NA segments (Figure 2C), and PB2 and NS segments (Figure 2D) were plotted. For the PDCP construction, all sequences were divided into all possible pairs. In the next step, the percentages of different nucleotides for the different genomic regions were calculated (axes in Figure 2), which determined the positions of the dots in the PDCP. It should be noted that virus pairs with different percentages of different nucleotides in different genomic regions are assumed to contain reassorted viruses. For example, the dots in the lower-right panel of Figure 2B–D indicate the virus pairs that differ by more than 10% of their nucleotides in the PB2 segment sequences but differ by less than 5% of their nucleotides in the HA, NA, and NS segment sequences, respectively. As a negative control for reassortment, full genomic sequences of H5N8 viruses were concatenated and the pairwise evolutionary distances for odd and even positions of this artificial alignment were calculated in a pairwise manner (Figure 2A).

To identify cases of reassortment, pairs were selected that differed by more than 10% in the PB2 segment and by less than 5% in their HA, NA, and NS segments (indicated by green boxes in Figure 2). A list of virus pairs was then generated, with sequence comparisons resulting in the dots highlighted by green rectangles in Figure 2. The occurrence of each virus in the dataset of the above virus pairs was counted (Supplementary Table S2). To facilitate the analysis, the cells in the columns of the different lists were manually coloured with a gradient according to their number of counts. On the ‘Overview’ and ‘Counts’ sheets of Supplementary Table S2, a simple colour scheme was used in which the colours run in ascending order from red to yellow to green. A more frequent involvement of a virus in pairs whose sequences show traces of phylogenetic incongruence when compared may indicate a direct involvement of the ancestors of these viruses in a reassortment event. Therefore, the selection of sequences for subsequent detailed analysis was determined by this factor.

On the “Distance” sheets, the columns “X” and “Y”, which indicate the genetic distances from the corresponding plots in Figure 2, were coloured according to the following rules: for column “X”, the cell with the smallest number was coloured red, the cell with the largest number was coloured green, and the values in between were coloured in ascending order along a gradient from red to green. The cells in column “Y” are coloured according to a similar rule, with the exception that the cell with the smallest number is coloured green and the cell with the largest number is coloured red.

**Figure 3:**
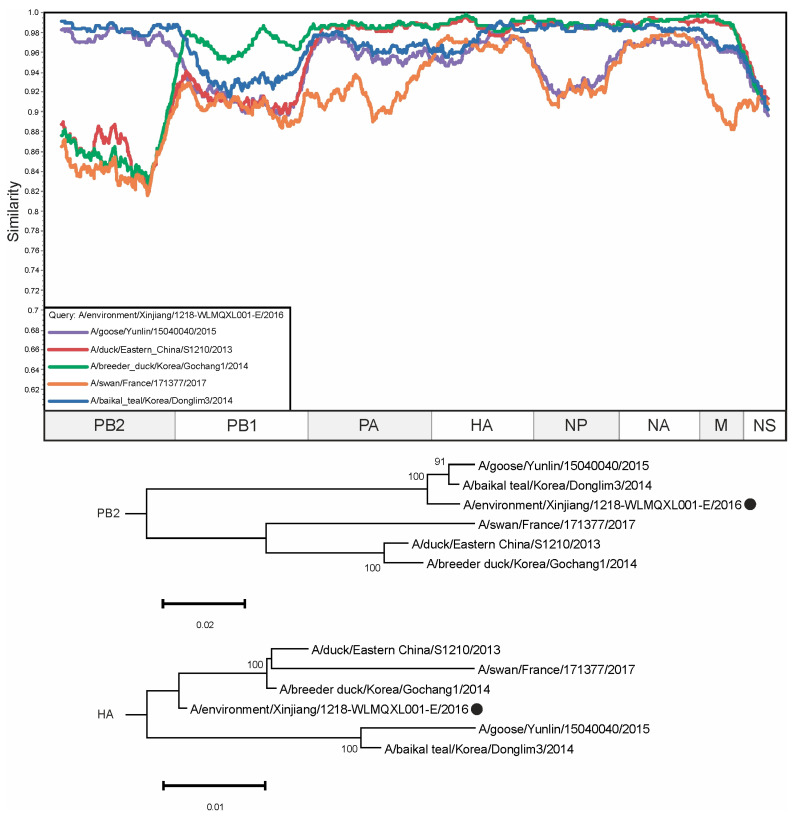
A similarity plot analysis (window = 600, step = 10) was performed. The genome of A/environment/Xinjiang/1218-WLMQXL001-E/2016 was selected as the query sequence. The *x*-axis shows the nucleotide position in the alignment and the *y*-axis shows the percentage similarity between the query sequence and five other selected viruses. Coordinates were found for each segment in the alignment and the segments were plotted on the *x*-axis of the similarity plot. In addition, phylogenetic trees were created for PB2 and HA segments. The black dot indicates the virus that was used as a query. The different trees show different topologies—the reliably supported nodes in the tree vary. Bootstrap support was indicated for nodes with support above 70.

**Figure 4:**
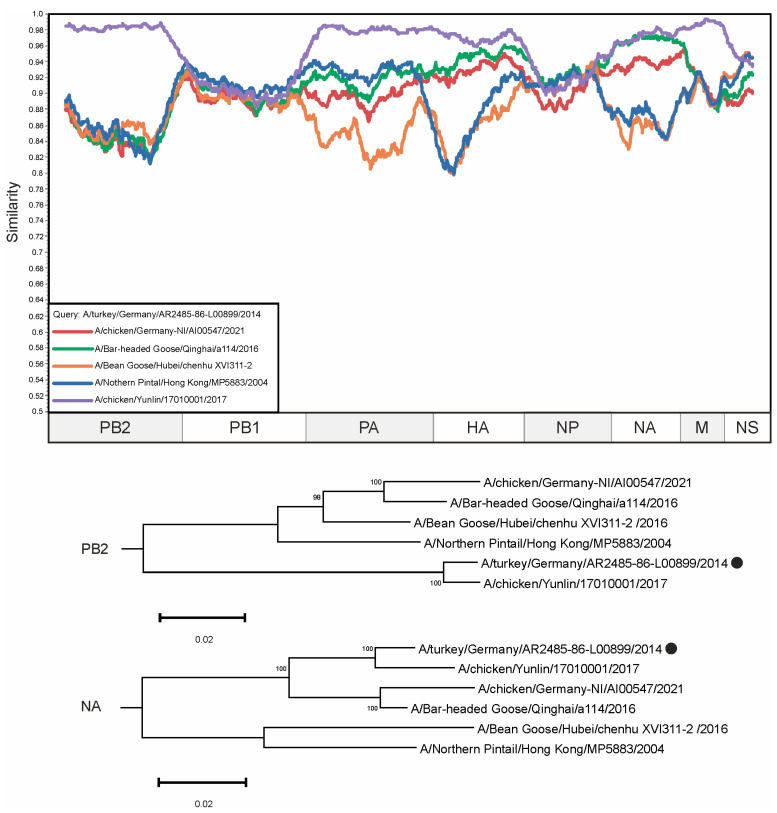
A similarity plot analysis (window = 600, step = 10) was performed. The genome of A/turkey/Germany/AR2485-86-L00899/2014 was selected as the query sequence. The *x*-axis shows the nucleotide position in the alignment and the *y*-axis shows the percentage similarity between the query sequence and five other selected viruses. Coordinates were found for each segment in the alignment and the segments were plotted on the *x*-axis of the similarity plot. In addition, phylogenetic trees were created for PB2 and NA segments. The black dot indicates the virus that was used as a query. The different trees show different topologies—the reliably supported nodes in the tree vary. Bootstrap support was indicated for nodes with support above 70.

**Figure 5:**
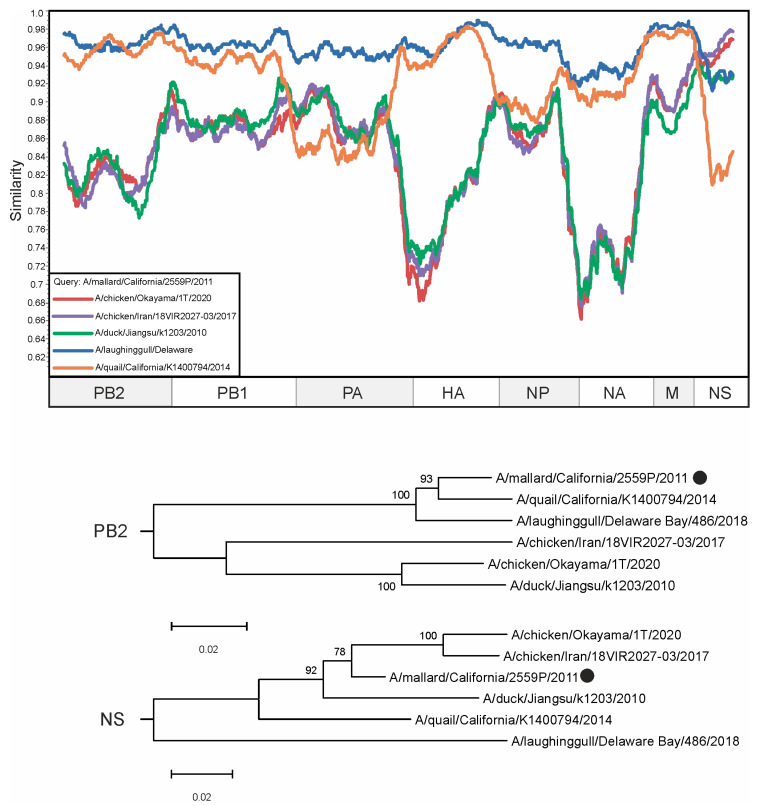
A similarity plot analysis (window = 600, step = 10) was performed. The genome of A/mallard/California/2559P/2011 was selected as the query sequence. The *x*-axis shows the nucleotide position in the alignment and the *y*-axis shows the percentage similarity between the query sequence and five other selected viruses. Coordinates were found for each segment in the alignment and the segments were plotted on the *x*-axis of the similarity plot. In addition, phylogenetic trees were created for PB2 and NA segments. The black dot indicates the virus that was used as a query. The different trees show different topologies—the reliably supported nodes in the tree vary. Bootstrap support was indicated for nodes with support above 70.

In total, 18,726 virus pairs were identified that showed signs of possible reassortment. The three most prevalent viruses in this dataset—A/environment/Xinjiang/1218-WLMQXL001-E/2016, A/turkey/Germany/AR2485-86-L00899/2014, and A/mallard/California/2559P/2011—were selected for further analysis. The concatenated sequences of these viruses were complemented with other concatenated virus genomes to reveal the patterns in the similarity plots (Figure 3, Figure 4 and Figure 5). In addition to the construction of similarity plots, phylogenetic trees were also created to validate the reassortment events.

Previously undescribed reassortment events were found in several H5N8 viruses. For example, the sequence of the PB2 segment of the virus A/environment/Xinjiang/1218-WLMQXL001-E/2016 is closer to those of the viruses A/baikal teal/Korea/Donglim3/2014 and A/goose/Yunlin/15040040/2015 (98.68% and 97.93% identical nucleotides, respectively), while the HA segment has 97.41% and 96.47% identical nucleotides, respectively. At the same time, the HA segment of A/environment/Xinjiang/1218-WLMQXL001-E/2016 shared 99.00% and 98.65% identical nucleotides with A/breeder duck/Korea/Gochang1/2014 and A/duck/Eastern China/S1210/2013, which had only 87.34% and 87.91% identical nucleotides in the PB2 segment, respectively (Figure 3). It should be noted that the HA segment of A/environment/Xinjiang/1218-WLMQXL001-E/2016 is most similar to the HA segments of viruses collected in East Asia (1.0–1.8% of differing nucleotides, Supplementary Table S2). Considering the high level of substitutions in H5N8 IAVs, it can be assumed that the reassortment occurred several years before the collection of the above viruses in East Asia.

The PB2 segment of A/turkey/Germany/AR2485-86-L00899/2014 was a clear outgroup for other viruses in the alignment (from 86.64% to 87.43% identical nucleotides), except for the sequence of virus A/chicken/Yunlin/17010001/2017, which was identical in 98.38% of its nucleotides. However, the situation was drastically different for the other segments. For example, the NA segment of A/turkey/Germany/AR2485-86-L00899/2014 shared 94.79% and 93.42% identical nucleotides with the NA segments of A/Bar-headed Goose/Qinghai/a114/2016 and A/chicken/Germany-NI/AI00547/2021, respectively (Figure 4). Interestingly, the HA segment of A/turkey/Germany/AR2485-86-L00899/2014 is most similar to the HA segments of viruses collected in Taiwan (1.6–2.0% differing nucleotides, Supplementary Table S2). However, in the absence of similar sequences of viruses collected in regions between Taiwan and Germany, it is hardly possible to draw conclusions about the history of the emergence and spread of the reassortant virus.

The sequence of the PB2 segment of the virus A/mallard/California/2559P/2011 has the highest similarity with that of the A/quail/California/K1400794/2014 virus sequence (96.72% identical nucleotides). At the same time, its NS segments shared 91.84% identical nucleotides, whereas the NS segment of A/mallard/California/2559P/2011 had 93.73% identical nucleotides with the NS segment of A/chicken/Okayama/1T/2020 and 94.56% identical nucleotides with the NS segment of A/chicken/Iran/18VIR2027-03/2017 (Figure 5). The NS segment of A/mallard/California/2559P/2011 is most similar to the NS segments of viruses collected in Iran and East Asia (2.9–3.8% differing nucleotides, Supplementary Table S2). It should be noted that there were no viruses in the analyzed dataset whose NS segment contained less than 2.9% different nucleotides with A/mallard/California/2559P/2011, which makes it difficult to correctly decipher this reassortment event.

**Figure 6:**
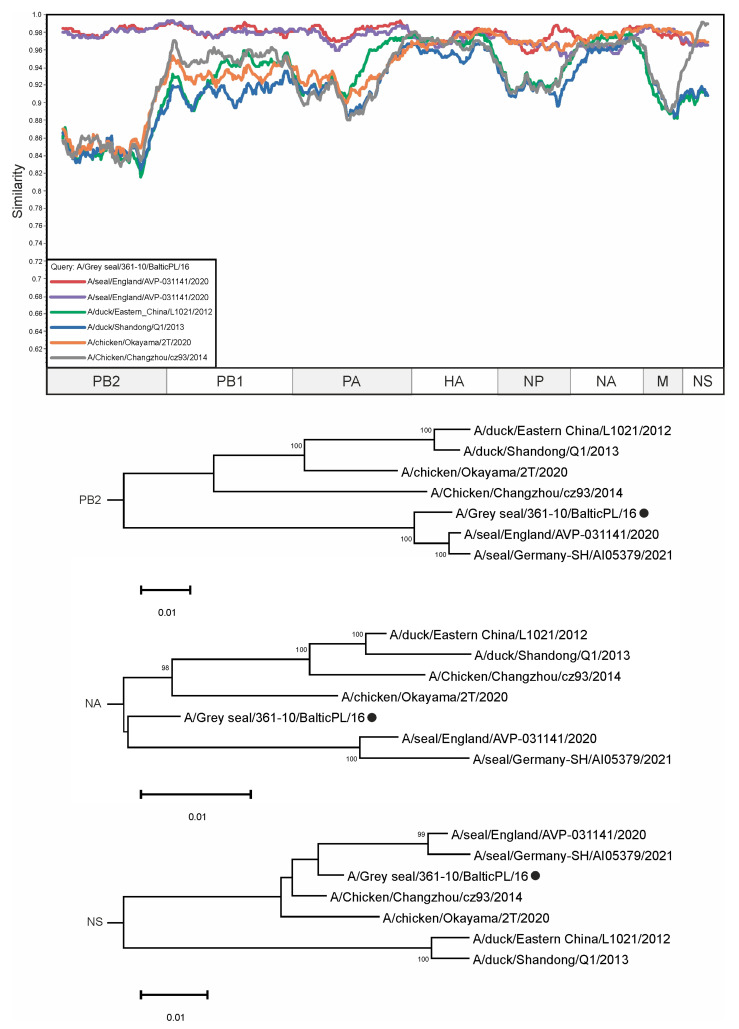
A similarity plot analysis (window = 600, step = 10) was performed. The genome of A/Grey seal/361-10/BalticPL/16 was selected as the query sequence. The *x*-axis shows the nucleotide position in the alignment and the *y*-axis shows the percentage of similarity between the query sequence and six other selected viruses. Coordinates were found for each segment in the alignment and the segments were plotted on the *x*-axis of the similarity plot. In addition, phylogenetic trees were created for the PB2 and NA segments. The black dot indicates the virus that was used as a query. The different trees show different topologies—the reliably supported nodes in the tree vary. Bootstrap support was indicated for nodes with support above 70.

The dataset contained three mammalian H5N8 influenza viruses: A/seal/England/AVP-031141/2020, A/seal/Germany-SH/AI05379/2021, and A/grey seal/361-10/BalticPL/16 (Figure 6). In the PB2 segment, the sequence of virus A/grey seal/361-10/BalticPL/16 has the highest degree of similarity to other seal viruses, namely A/seal/England/AVP-031141/2020 and A/seal/Germany-SH/AI05379/2021—having 98.33% and 98.11% identical nucleotides, respectively. In comparison, the sequence of the virus A/chicken/Okayama/2T/2020 has a relatively lower degree of similarity for the PB2 segment—87.78%—and belongs to the group of viruses collected in East Asia (Figure 6, upper phylogenetic tree). At the same time, the phylogenetic relationship based on the NA segment sequence shows a different pattern. The sequence of A/chicken/Okayama/2T/2020 has about the same number of identical nucleotides as those of A/duck/East China/L1021/2012, A/grey seal/361-10/BalticPL/16, A/seal/England/AVP-031141/2020, A/seal/Germany-SH/AI05379/2021—95.90%, 96.32%, 95.90%, and 95.13%, respectively. With regard to the NS segment of the virus A/grey seal/361-10/BalticPL/16, the sequence of the virus A/Chicken/Changzhou/cz93/2014 is the most similar, with an identity of 98.87%. The viruses A/seal/England/AVP-031141/2020 and A/seal/Germany-SH/AI05379/2021 have 97.74% and 97.28% of their nucleotides in common with A/Grey_seal/361-10/BalticPL/16, respectively, based on the NS segment sequence.

## 4. Discussion

To date, the H5N8 viruses have spread throughout Eurasia and North America due to the seasonal migration of birds. The occurrence of the highly pathogenic avian influenza virus is being monitored in numerous countries worldwide. In 2020, for example, a new reassortant, Ger-01-20, was discovered in two HPAIV viruses isolated from dead birds in Germany. This reassortant had six segments that were highly similar to H5N8 viruses from Asia, Europe, and Africa and two segments that resembled a low pathogenic H3N8 virus from Russia, suggesting significant genetic diversity and reassortment [38]. Another notable study examined 63 H5N8 isolates from Japan in the winter of 2020–2021 and identified two clusters (G1 and G2) within clade 2.3.4.4b. Phylogenetic analysis revealed that these isolates arose from a reassortment between avian influenza viruses from Siberia and Europe, with G1 viruses spreading from Europe to Eurasia and G2 viruses first spreading in Siberia before spreading to Europe and Asia [39]. Reassortant H5N8 viruses therefore emerge regularly, which is dangerous in terms of a pandemic threat, as there are no approved vaccines that can prevent the disease. According to information from ClinicalTrials.gov, there are four clinical trials in the field of the H5N8 vaccine, two of which are in the final phase, while the others are in the active phase [40].

In this study, all available H5N8 influenza virus sequences in the GISAID database were analyzed for reassortment events within a subtype. With 2359 viral sequences as the input, many potential reassortment events (Supplementary Table S2) were identified. After selecting a small subset of potential reassortants (green boxes in Figure 2), we used similarity plots and phylogenetic trees for different segments to find novel viruses whose occurrence had not been previously described. The different tree topologies indicate the complex evolutionary history of each virus.

During the analysis, several known reassortment viruses were identified that could provide a positive control for the automatic reassortment search approach. Phylogenetic inconsistencies were found in a group of viruses from Japan (for HA, NA, and NS segments) (Supplementary Figure S1) that were previously described as reassorting [39]. Another previously described case of reassortment, evidenced by a patent describing the creation of a new chimeric influenza virus strain for vaccine production, was also found (Supplementary Figure S2) [32]. Our results are consistent with those of the aforementioned study, suggesting that this approach is effective in detecting reassortment phenomena. In addition, reassortment was found in viruses collected from marine mammals (Figure 6) [30,31,32,33,34,35,36,37,38,39,40,41]. These events prove that reassortant forms of influenza H5N8 are capable of infecting mammals and pose a serious threat to humans. In this case, no other viruses involved in the reassortment process described in Figure 6 were found in the regions between East Asia and the Baltic Sea coast; therefore, no conclusions can be drawn about the possible mechanism for the emergence of reassortant viruses in European marine mammals. This is most easily explained by the incomplete knowledge of the actual diversity of H5N8 on a global scale

In the course of the analysis, we discovered an uneven geographical distribution of the related IAVs. An illustrative example is A/mallard/California/2559P/2011, whose similar segment can also be found in the viruses A/chicken/Iran/18VIR2027-03/2017 and A/chicken/Okayama/1T/2020. It should be noted that there are other regions between the places of the viruses’ collection where there were relatives of these viruses whose sequences are not yet known. The observed inconsistencies in the geographical spread of viruses can be traced back to their main vectors, birds. They often migrate from one geographic region to another in search of food and other resources. They also participate in seasonal migrations where they travel long distances [42]. In such cases, the co-infection of birds with multiple viruses is possible, which can lead to reassortment [17]. Effective surveillance for HPAI is only a partial solution in some countries as there are no border controls for birds. Therefore, knowledge about the actual spread of viruses with high pandemic potential appears to be fragmentary. Based on these considerations, the global surveillance system is a promising approach to overcome the challenges of “gray areas” [43].

The process of reassortment determines, to a certain extent, how viruses evolve and adapt and how new strains emerge that can cause disease outbreaks [44,45]. Monitoring new reassortants enables their timely detection and, accordingly, the optimization of vaccine formulation to maintain their efficacy. Understanding how reassortant viruses spread among humans and animals also helps in assessing the risk of pandemics and developing strategies to prevent them. A new influenza strain emerged by reassortment can become more virulent or, conversely, have a weakened pathogenicity [46]. These changes need to be detected as early as possible.

Herein, we have revealed several cases of potential reassortments (Figure 3, Figure 4 and Figure 5) that were not previously described. At the same time, our prediction suggests that there are a large amount of other undescribed viruses that may have arisen as a result of reassortment. The pairs of such candidate reassortment viruses are listed in Supplementary Table S2. A total of 18,726 occurrences were identified, of which the most frequent were selected for detailed analysis (Figure 3, Figure 4 and Figure 5).

## 5. Conclusions

This comprehensive analysis of H5N8 influenza virus sequences has revealed significant reassortment events that have led to the exchange of different virus segments. These results underscore the dynamic nature of influenza virus evolution and the ongoing threat posed by highly pathogenic avian influenza viruses. Effective surveillance and a deeper understanding of reassortment mechanisms are crucial for the prediction and containment of potential influenza pandemics. This study provides valuable insights into the evolutionary pathways of H5N8 viruses and highlights the need for further research and surveillance to prevent future outbreaks.

## Data Availability

Restrictions apply to the availability of these data. Data were obtained from GISAID and are available at https://gisaid.org/ (accessed on 18 December 2023)with the permission of GISAID.

## Supplementary Materials

The following supporting information can be downloaded at: https://www.mdpi.com/article/10.3390/v16091405/s1, Supplementary Table S1: Meta information on the sequences used, Supplementary Table S2: Analysis of genetic distances between PB2, HA, NA and NS viruses segments received during construction PDCP, highlighted by green boxes on Figure 2; Figure S1: Similarity plot for group of Asian viruses and phylogenetic trees for PB2, HA, NA and NS segments, Figure S2: Similarity plot analysis of A/UNL/HK/2:6/2017 (H5N8)).
