# Supplementary material for: Complex Evolutionary Dynamics of H5N8 Influenza A Viruses Revealed by Comprehensive Reassortment Analysis
Source: Viruses. 2024 Sep 3;16(9):1405. doi: 10.3390/v16091405 (PMC11437431; doi:10.3390/v16091405)
Supplement: Supplementary file 1 [file viruses-16-01405-s001.zip › Supplementary Figure_S1.pdf]

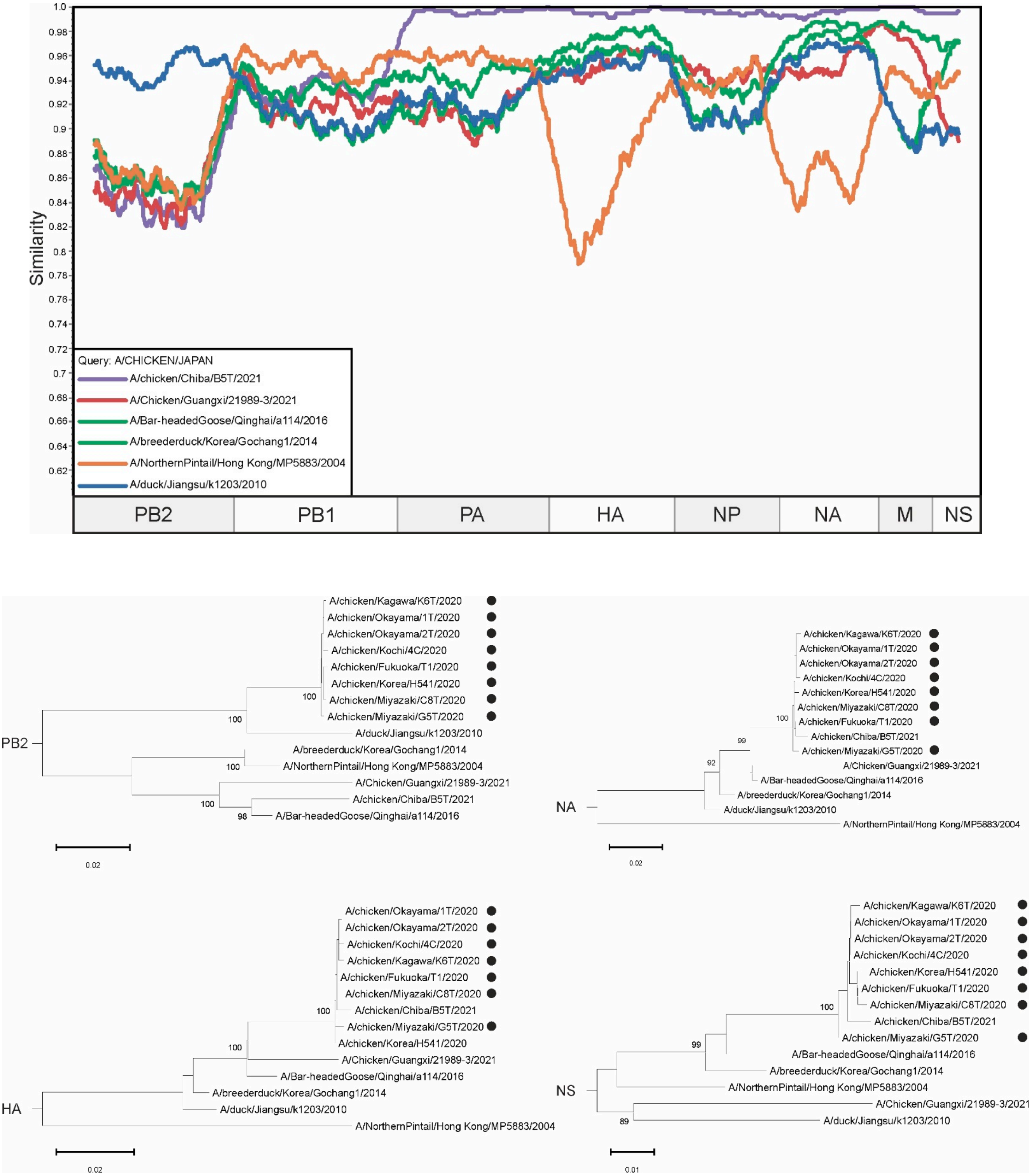

Supplementary Figure S1. . A similarity plot analysis (window = 600, step = 10) was performed. Query is represented by the 50% consensus of genomes of A/chicken/Fukuoka/T1/2020, A/chicken/Kagawa/K6T/2020, A/chicken/Kochi/4C/2020, A/chicken/Korea/H541/2020, A/chicken/Miyazaki/C8T/2020, A/chicken/Miyazaki/G5T/2020, A/chicken/Okayama/1T/2020, A/chicken/Okayama/2T/2020.The x-axis shows the nu-cleotide position in the alignment and the y-axis shows the percentage similarity between the query sequence and five other selected viruses. Coordinates were found for each segment in the alignment and the segments were plotted on the x-axis of the similarity plot. In addition, phylogenetic trees were created for PB2, HA, NA and NS segments. The black dot indicates the virus that was

used as a query. The different trees show a different topology - the reliably supported nodes in the tree vary. Bootstrap support was indicated for nodes with support above 70
