# Supplementary material for: Complex Evolutionary Dynamics of H5N8 Influenza A Viruses Revealed by Comprehensive Reassortment Analysis
Source: Viruses. 2024 Sep 3;16(9):1405. doi: 10.3390/v16091405 (PMC11437431; doi:10.3390/v16091405)
Supplement: Supplementary file 1 [file viruses-16-01405-s001.zip › Supplementary Figure_S2.pdf]

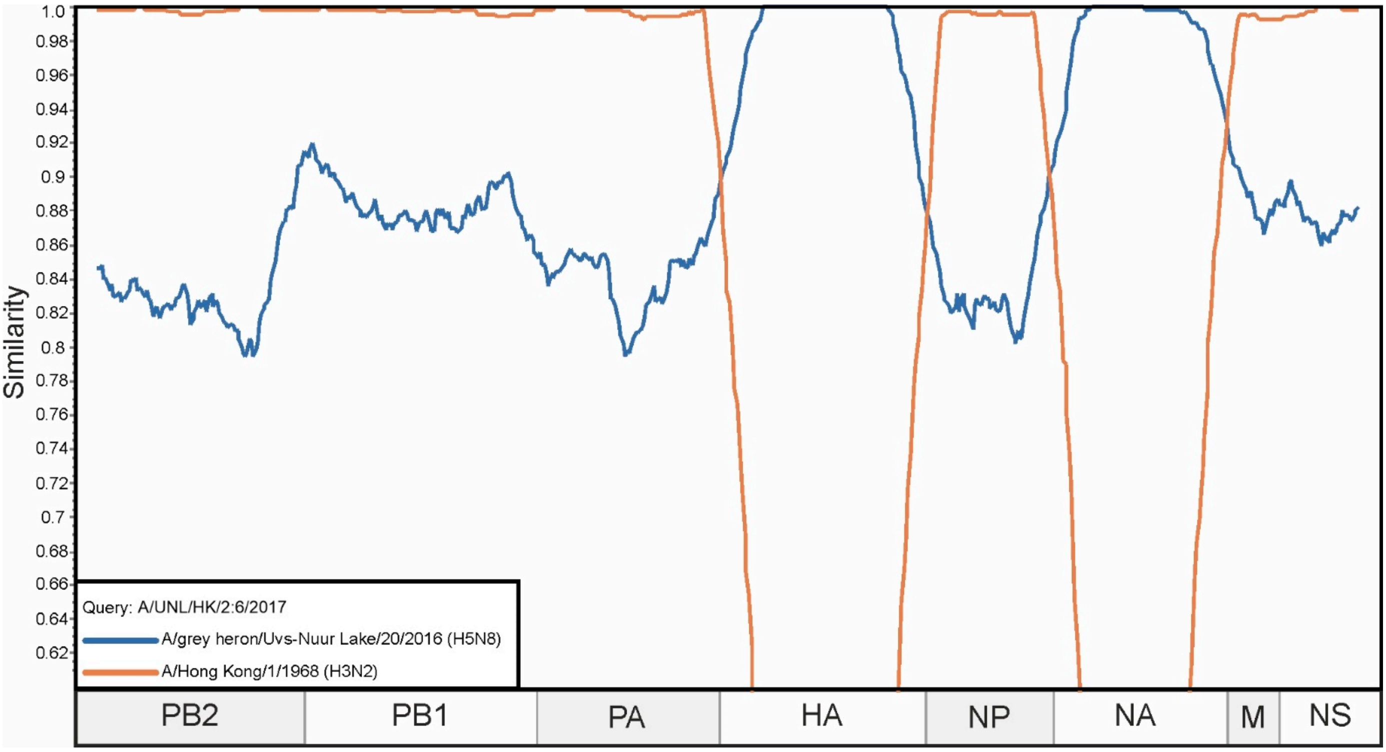

Supplementary Figure S2. A similarity plot analysis (window = 600, step = 10) was performed. The genome of A/UNL/HK/2:6/2017 was selected as the query sequence. The x-axis shows the nucleotide position in the alignment and the y-axis shows the percentage similarity between the query sequence and two other selected viruses. Coordinates were found for each segment in the alignment and the segments were plotted on the x-axis of the similarity plot
